# Supplementary figures and images for: A high resolution atlas of gene expression in the domestic sheep (Ovis aries)
Source: PLoS Genet. 2017 Sep 15;13(9):e1006997. doi: 10.1371/journal.pgen.1006997 (PMC5626511; doi:10.1371/journal.pgen.1006997)

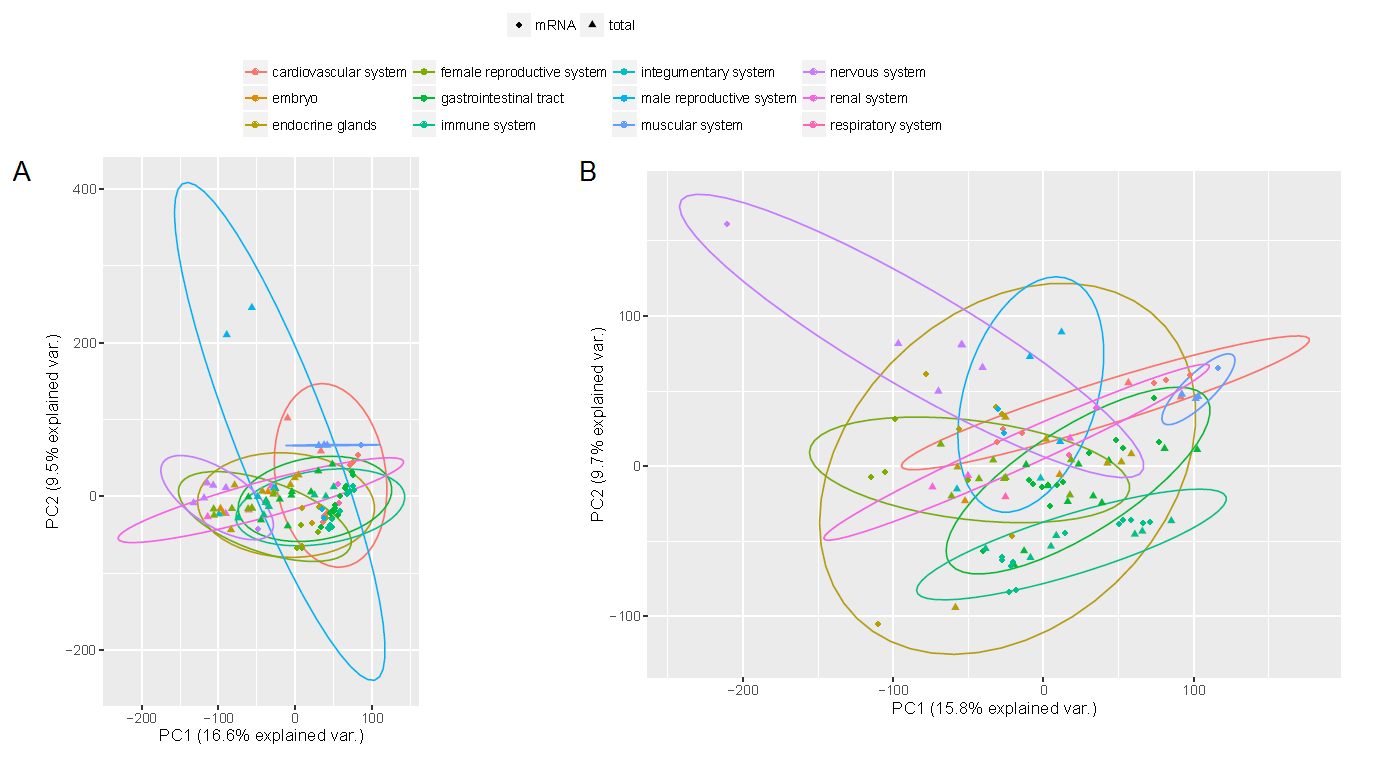

Supplement: S1 Fig — For each sample, each gene’s expression level is taken as the mean TPM across all replicates, before (A) and after (B) any batch effect correction. Samples are coloured by organ system. Ellipses indicate confidence intervals of 95%. The shape of each point indicates each sample’s library type: mRNA-seq (circle) or total RNA-seq (triangle). Blastocyst samples are excluded for clarity as they are generated using a different experimental protocol. Before correction, points can be partitioned by shape (to the left and right of sub-Fig A), suggesting a batch effect–variation introduced by library type confounds variation by tissue type. After correction (sub-Fig B), there is no notable axis of variation that partitions points by shape–consequently, variation introduced by library type (a batch effect) does not confound variation by tissue type (which is biologically meaningful). (TIFF) [file pgen.1006997.s001.tiff]

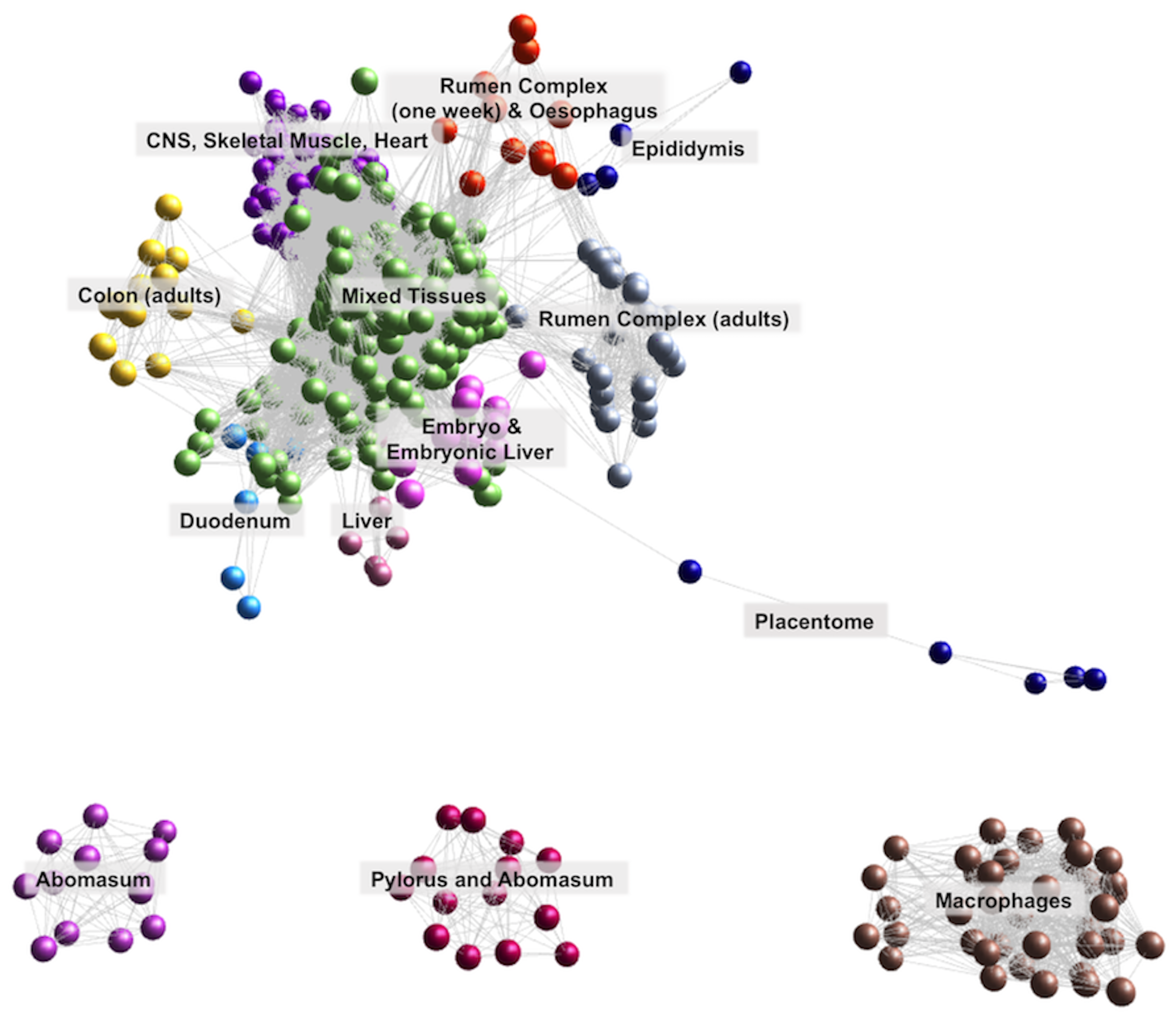

Supplement: S2 Fig — Each node represents a sample and each edge its connectivity to other samples in the dataset. A correlation of r = 0.75 split the graph into 10 different clusters. The largest cluster (cluster 1) included the majority of samples (‘mixed tissues’), most of which were transcriptionally similar, while the remainder of the clusters comprised samples with distinctive transcriptional signatures such as macrophages (3) and abomasum. Spurious samples were easily identified if they were present in a cluster comprised of samples from a different tissue or cell type. Pearson correlation r = 0.75, MCLi = 2.2, nodes = 481 and edges = 23,903. (TIFF) [file pgen.1006997.s002.tiff]
