# Supplementary material for: A high resolution atlas of gene expression in the domestic sheep (Ovis aries)
Source: PLoS Genet. 2017 Sep 15;13(9):e1006997. doi: 10.1371/journal.pgen.1006997 (PMC5626511; doi:10.1371/journal.pgen.1006997)
Supplement: S1 Methods — (DOCX) [file pgen.1006997.s003.docx]

**S1 Methods – Additional Methods**

This document provides additional detail on the methodology used to create and annotate the sheep expression atlas. The ‘two-pass’ method of alignment-free atlas creation, along with the means by which the effects of library type upon expression level are corrected, are discussed more fully in a satellite publication [1]. Some of this material is recapitulated here.

**An alignment-free approach to expression atlas creation**

The conventional means by which RNA-Seq data is processed is to align the sequenced reads to a reference genome, to reconstruct transcripts from this set of alignments and to quantify their expression as a function of the reads aligned [2]. Successors to this approach have been devised in the form of ‘lightweight’ or alignment-free algorithms (such as Sailfish [3], Salmon [4], RNA-skim [5] and Kallisto [6]), which – by simplifying the nature of the task – achieve large increases in processing speed without loss of accuracy. Kallisto, for instance, builds an index of k-mers from a known set of transcripts and estimates expression level from the reads directly – rather than aligning reads to each transcript (a time-consuming approximation, as alignments have gaps), k-mers (generated from transcripts) are instead matched exactly to each read [6]. This generates expression level estimates orders of magnitude faster than previous approaches, and so is in principle ideal for processing the large volumes of data constituting an expression atlas.

Nevertheless, Kallisto is not without limitations. By relying upon a reference transcriptome (rather than a genome), it cannot reconstruct novel transcripts or splice junctions, nor revise existing gene or transcript models. Furthermore, alignments – by definition – assign reads to specific locations within a transcript and so are robust to sequencing errors or low-quality regions of the reference. By contrast, Kallisto identifies only which transcript generated a read, not where in the transcript it did so. Accordingly, alignment-free approaches are likely to be inaccurate when used with a poor-quality index. Such an index will be more likely to have missing transcript models (which Kallisto will not detect) and miscalled bases (such that k-mers will not exactly match the reads, skewing each transcript’s expression level estimate).

Kallisto is thus contingent on the presence of a reliable reference transcriptome. In principle, this could be derived from the reads directly, by performing a genome-guided *de novo* assembly. However, this would effectively trade one computational bottleneck for another – Kallisto is quick to quantify expression (~30 million reads can be processed in <10 minutes [6]) but we can only be confident of its estimates if we have an accurate reference transcriptome, the creation of which is slow (for instance, 4 million reads can be processed in ~60-90 minutes by the Trinity assembler [7]). We resolve this issue with a ‘two pass’ approach. This involves pseudoaligning all reads against the complete reference transcriptome (the first pass [of data through Kallisto]), parsing this output to create a revised version, and then quantifying expression using that revised reference (the second pass).

First, we run Kallisto v0.43.0 [6] on all samples, using the --pseudobam parameter and, as its k-mer index (k=31), the Oar v3.1 reference transcriptome (ftp://ftp.ensembl.org/pub/release83/fasta/ovis_aries/cdna/Ovis_aries.Oar_v3.1.cdna.all.fa.gz; n=23,113 transcripts [22,823 protein-coding, 247 pseudogene, 43 processed pseudogene], pooled together with n=6005 cDNAs for non-protein coding transcripts, obtained from Ensembl BioMart: lincRNA, miRNA, misc_RNA, Mt_rRNA, Mt_tRNA, rRNA, snoRNA, snRNA). Taken together, this represents 27,054 genes.

We then parse the resulting data (approx. 26 billion pseudoalignments; see **S3** **Table**) so as to revise this index. This is in order to include, in the second index, those transcripts that are absent from the original (i.e., where the reference annotation is incomplete), and to exclude misleading or incorrect reference transcripts (i.e., where the reference annotation is poor quality and a spurious model has been introduced). For the first criterion, we obtain the subset of reads that Kallisto could not align, assemble these *de novo* into putative transcripts, then retain each transcript only if it can be robustly annotated (by, for instance, encoding a protein similar to one of known function) (see below: ‘revision of the reference transcriptome’). For the second criterion, we identify those members of the reference transcriptome with zero expression across all samples. These are then discarded from the index. By doing so, we will increase the fraction of unique k-mers amongst the remaining transcripts in the index, in turn improving the accuracy of Kallisto’s estimated counts.

Nevertheless, we cannot discard alternative explanations for the absence of detectable expression, although consider them unlikely: unexpressed transcripts could instead be highly tissue-specific (for a tissue not sampled in this study) or so lowly expressed that we cannot detect them at the sampling depths used.

In finalising the revised reference transcriptome, we also restrict data only to protein-coding genes, pseudogenes and processed pseudogenes. All non-coding transcripts – abundant in the total RNA-Seq but not the mRNA-Seq libraries – are excluded so that Kallisto will estimate expression level for an equivalent RNA space per sample. The revised reference transcriptome contains 23,115 transcripts, representing 21,218 genes.

A summary of the genes detectable using successive Kallisto indices is given in **S24 Table**.

**Revision of the reference transcriptome**

To create the ‘second pass’ index, we first identify those reads that Kallisto could not align (for each sample’s pseudobam file, using SAMtools v1.3 with parameter -f 4 [8]). For each sample, unmapped reads are then assembled *de novo* using the Trinity assembler version r20140717 [7, 9] (which also makes use of the k-mer counting algorithm Jellyfish v2.2.5 [10] and the aligner Bowtie v1.1.2 [11]).

We then filter these assembled transcripts to retain only those that can be robustly annotated, excluding those whose CDS is unlikely to encode a protein (as these are less likely to be real). The following criteria are applied: (a) the transcript must encode an ORF of at least 100 amino acids (using TransDecoder v2.1.0 [7] with LongOrfs parameters -S and -m 100), which (b) must contain a known protein domain (based on a search, by HMMER v3.1b2 [12] with E-value 1e-5, of the Pfam database of protein families, v29.0 [13]), and (c) must share homology with a known peptide (based on a search, by BLAST+ v2.3.0 [14], of the Swiss-Prot [15, 16] March 2016 release: blastp [17] with parameters -max_target_seqs 1 and -evalue 1e-10). We filter the results to remove those Swiss-Prot entries that are protein fragments, and those whose PE (protein existence) code is not either 1 or 2 (‘experimental evidence at the protein level’ [such as by Edman sequencing, mass spectrometry, X-ray or NMR structure, evidence of protein-protein interaction or antibody-based detection] and ‘experimental evidence at the transcript level’ [such as the existence of cDNA, RT-PCR or Northern blots], respectively).

We then associate, per sample, each CDS with its best hit Swiss-Prot accession: the hit with the longest alignment length, after excluding all hits with < 75% identity. This alignment is validated using the ‘needle’ module of the EMBOSS suite [18] with parameters -gapopen 10.0 and -gapextend 0.5 (‘needle’ implements the Needleman-Wunsch algorithm, i.e. provides a global [end-to-end] alignment rather than the local alignment of blastp). Global alignments are made between the peptide encoded by the predicted ORF and its best Swiss-Prot hit. Those that have < 75% identity are discarded.

Gene symbols are then assigned according to the best Swiss-Prot hit, unless that symbol is already present in the reference annotation (or one of its synonyms [according to NCBI: ftp://ftp.ncbi.nlm.nih.gov/gene/DATA/gene_info.gz, downloaded 7^th^ April 2016]).

Finally, we assess these sequences for coding potential using the online tool CPAT v1.2.2 [19] (http://lilab.research.bcm.edu/cpat/index.php, accessed 12^th^ April 2016). CPAT assigns a coding probability to sequences based on both Fickett TESTCODE score (which distinguishes protein-coding RNA from ncRNA according to nucleotide composition and codon usage bias) [20] and differential hexamer usage (which, given the dependence between adjacent amino acids in a peptide, discriminates coding from non-coding sequences with high accuracy) [21]. CPAT significance cut-offs were applied based on the human hg19 assembly (as the sheep assembly is not explicitly supported). After excluding those with low coding probability (in the case of a human model, if p < 0.364), 49 sequences were retained (**S23** **Table**). These are indicative of novel transcripts.

**Correcting expression level estimates for the effect of library type**

By default, Kallisto outputs TPM (transcripts per million) estimates, a normalised measure of relative transcript abundance comparable within, but not between, samples [22]. This is because the abundance of one transcript affects the relative abundance of all others (for instance, if in one tissue a gene is more highly expressed, the reported TPM of all other genes will be lower, irrespective of their absolute abundance).

However, meaningful comparisons between data generated by the two different experimental protocols – poly(A)-selected (mRNA-Seq) and rRNA-depleted (total RNA-Seq) – cannot be made without applying a correction. This is because rRNA-depleted libraries contain both poly(A)+ and poly(A)- transcripts whereas poly(A)- selected libraries, by definition, are restricted to the latter. rRNA-depleted libraries also have a comparatively high number of reads mapping to non-coding regions, and so a relative overabundance of intronic transcripts [23]. As such, if we were to directly compare poly(A)- and rRNA-depleted data, the expression levels of protein-coding genes in the latter would be lower: exonic sequencing depth is artificially reduced if the number of intronic reads is comparatively high (which occurs with TRIzol RNA extraction) [23]. The two protocols have effectively selected different fractions of the same transcriptome – a million reads from an mRNA-Seq library are not equivalent to a million reads from a total RNA-Seq library [24-26].

We corrected for this effect as follows. First, we estimate expression level for a standardised RNA space for each sample (the ‘second pass’ index). Secondly, we calculated, per gene, the ratio of mean TPM across all mRNA-Seq libraries to mean TPM across all total RNA-Seq libraries. Given the scope of the tissues sampled for both library types (all major organ systems from both sexes and from different developmental states), neither mean is likely to be skewed by any tissue-specificity of expression. As such, any deviations of this ratio from 1 will reflect variance introduced by library type/depth. Thus, to correct each gene’s set of expression estimates for this batch effect, we multiplied all total RNA-Seq TPMs by this ratio.

A principal component biplot of the corrected TPM estimates demonstrates a biologically meaningful distribution of data – samples group appropriately by organ system irrespective of library type (**S1 Fig**). An additional validation of the data may be made using hierarchical clustering (as in [27]), visualising the distribution of expression estimates in a manner formally representing a phylogenetic tree (**Fig 1**). Groups established by expression similarity are in many cases functionally or compositionally similar [28]: for example, related macrophage cell lines (alveolar, monocyte-derived and bone marrow-derived) cluster together, as do lymph nodes, and both auricles and ventricles can be found within a ‘cardiac’ cluster.

This method is discussed in further detail in [1].

**Categorising expression profile**

To provide an at-a-glance interpretation of the atlas, we categorise all genes using a scheme employed in the Human Protein Atlas [29]. Firstly, genes can be considered to have high, medium or low mRNA abundance according to the number of tissues/cell lines in which there is detectable expression. Abundance is determined by TPM thresholds, such that (a) no detectable expression: TPM < 1, (b) low expression: TPM >= 1 and < 10, (c) medium expression: TPM >= 10 and < 50, (d) high expression: TPM >= 50.

Genes are also assigned one or more categories as follows: (a) ‘tissue enriched’ (expression in one tissue at least five-fold higher than all other tissues [‘tissue specific’ if all other tissues have 0 TPM]), (b) ‘tissue enhanced’ (five-fold higher average TPM in one or more tissues compared to the mean TPM of all tissues with detectable expression [this category mutually exclusive with ‘tissue enriched’), (c) ‘group enriched’ (five-fold higher average TPM in a group of two or more tissues compared to all other tissues [note that all groups in this study – organ systems – contain two or more tissues with the exception of the respiratory system, for which data is available only for the lung]), (d) mixed expression (detected in one or more tissues and neither of the previous categories), (e) ‘expressed in all’ (>= 1 TPM in all tissues), and (f) ‘not detected’ (< 1 TPM in all tissues). These categories are based on simple thresholds so as to allow an overview of the data only, and are not intended to have statistical support.

**Gene annotation**

Of the 20,921 protein-coding genes in the Oar v3.1 annotation, 6217 (~30%) have only an Ensembl placeholder ID, rather than an HGNC name. These are annotated by reference to the NCBI non-redundant (nr) peptide database v77 [30] (which contains 94,391,349 sequences including GenBank CDS translations and entries from the Protein Data Bank/rcsb.org, SwissProt/uniprot.org, Protein Information Resource/pir.georgetown.edu, and the Protein Research Foundation/prf.or.jp). For each unannotated gene, we take the longest encoded peptide and obtain the set of blastp alignments [17] against NCBI nr, at a scoring threshold of p <= 1e^-25^. The set of alignments constitute a repository of possible gene descriptions, of which one can be selected as an annotation for that gene. If doing so, we also assign a numerical quality category of 1 to 8 for each annotation (1 being the highest quality). These categories represent incremental improvements in the informative content of the set of alignments (illustrated in **S5 Table**). The lowest quality category, 8, is simply the blastp hit with the lowest E-value. All subsequent quality categories require higher-quality hits that: (a) have a % identity within the aligned region of >= 90%, (b) have an alignment length >= 90% of the length of the query protein, (c) have an alignment length >= 50 amino acids, (d) have no gaps, and (e) are not to a protein labelled either ‘low quality’, ‘hypothetical’, ‘unnamed’, ‘uncharacterized’ or ‘putative’, or otherwise having a third-party annotation (as these can be by inference, not experiment). Quality category 7 is the best-scoring (i.e. lowest E-value) of these filtered ‘higher quality’ alignments. Category 6 is as above, but with at least one identifiable hit to a RefSeq ID for either human or one of 9 known ruminant proteomes (**S7 Table**). Category 5 requires that the set of alignments span at least 4 different genera (excluding *Ovis*). At this point, if >= 75% of the alignments have the same description, the gene is named for the associated HGNC name (according to ftp://ftp.ebi.ac.uk/pub/databases/genenames/new/tsv/locus_types/gene_with_protein_product.txt, downloaded 24^th^ August 2016). However, as NCBI nr aggregates multiple sources of data, gene descriptions have numerous synonyms and so it is not always possible to automatically assign an HGNC symbol. Unannotated genes with an associated description, but no gene name (that is, genes whose annotation is categories 6 to 8), are listed in **S10 Table**.

The highest quality categories, 1 to 4, not only meet the above criteria but have degrees of reciprocal % identity to at least one ruminant proteome (**S7 Table**). The highest quality category, 1, is if there is also a near-perfect match to an existing, related, peptide (alignment length >= 90% of the length of a protein from one of the 9 ruminant proteomes). Other quality categories, in descending order, are: 2 (alignment length >= 75% of the length of a related ruminant protein), 3 (alignment length >= 50% of the length of a related ruminant protein), and 4 (alignment length < 50% of the length of a related ruminant protein).

For the highest-quality annotations (category 1), this reliably identifies copy number variants, such as – for instance – a GSTM1 (glutathione S-transferase mu 1) cluster orthologous to humans [31] (Ensembl IDs ENSOARG00000019281, ENSOARG00000019285, ENSOARG00000019297, and ENSOARG00000019307).

**Tissue specificity indices**

For each gene, we calculate three tissue specificity indices. The first, *tau*, is a scalar measure of how broadly expressed a gene is [32]. *Tau* is bounded between 0 (for housekeeping genes) and 1 (for genes expressed in a single tissue only). For each gene with a set of expression estimates *i_1_*, *i_2_*, ..., *i_n_*, then letting *x_i_* = expression level normalised by maximum expression level and *n* = number of tissues, then *tau* is calculated as:

$$Tau= \frac{\sum_{i=1}^{n} (1-x_{i})}{n-1}$$

Compared to other measures of tissue specificity (such as the entropy of a gene’s expression distribution, *H_g_* [33], or the otherwise unnamed index of [34]), the use of *tau* results in well-separated groups with lower skew towards calling genes as either ubiquitous or tissue-specific. This same study reports that many tissue specificity indices are skewed towards classifying genes as ubiquitous and in this respect, *tau* is a superior means of capturing variation in expression profile.

The second tissue specificity index is calculated, per gene, as the mean TPM (across all tissues) divided by the median TPM (across all tissues). Genes with greater tissue specificity in their expression profile will have a more strongly right-skewed distribution (higher mean, lower median); consequently, the greater this index, the more tissue-specific the expression. To prevent undefined values, we apply a correction of +0.01 in the event that median TPM = 0. Note that unlike *tau*, this index has no upper bound.

Finally, we calculate a preferential expression measure (PEM) [35]. Unlike *tau* and mean/median TPM, which summarise in one value a gene’s tissue specificity, PEM is calculated per tissue per gene. PEM quantifies the expression of any given gene in any given tissue in relation to its average expression in all tissues (for each tissue *t_i_*, PEM(*t_i_*) = S-A, where S = gene expression in the specific tissue *t_i_*, and A = arithmetic mean expression of the gene in all tissues). As there are biological replicates per tissue, we considered *S* to be the mean TPM per gene and *A* to be the mean of all values of *S*. Before PEM was calculated, all values < 1 were considered to be 1, and a log_2_-transformation then applied. Genes with expression indistinguishable from noise (TPM < 1) will have a PEM of 0. More tissue-specific genes will have higher PEM values.

**Alignment-based RNA-Seq processing**

To complement the alignment-free expression atlas, we generated a parallel dataset using an alignment-based processing pipeline. This data can be used both as a confirmatory validation of Kallisto’s expression estimates, and because alignment-based – unlike alignment-free – methods can be used to identify novel, and revise existing, gene and transcript models.

After screening with FastQC v0.11.2 [36], all raw reads were cleaned using Trimmomatic v0.35 [37] with parameters ‘TRAILING:20 SLIDINGWINDOW:4:20 MINLEN:100.’ These parameters, respectively, remove bases from the end of a read if they are below a Phred score of 20, clip the read if the average Phred score within a 4bp sliding window advanced from the 5’ end falls below 20, and specifies a minimum read length of 100bp (Phred scores are the logarithm of the probability that a base was called incorrectly, i.e., a score of 20 is equivalent to 99% accuracy). The parameter ‘HEADCROP:8’ was also used for the blastocyst samples as these were generated using the NuGen Ovation Single Cell RNA-Seq System (http://www.nugen.com/sites/default/files/M01363_v10 - User Guide, Ovation Single Cell RNA-Seq System.pdf).

These cleaned reads were then aligned against the reference genome (Oar v3.1) using HISAT2 v2.0.4 [38] with the parameter --dta (optimise for downstream transcriptome assembly) and default alignment scoring parameters. In brief, HISAT2 assigns scores to alignments equal to the sum of the scores for individual mates (i.e. two scores for paired-end alignments, one for single-end [unpaired] alignments). Reads are required to align in full and are scored according to successful matching and penalised for mismatching: +2 for each position where a base in the read exactly matches that of the reference, -1 for any ambiguous base (N) on either the read or the reference, -(5+3*n*) for any gap opening or extension (of length *n*) on either the read or reference, -(2 + floor(4 x min(*Q*,40)/40)) where *Q* is the Phred quality score for any non-N mismatch between the read and reference. The minimum alignment score for reporting is -18. If there are a set of multiple valid alignments, the primary alignment is considered the one whose score is greater than or equal to any other member of the set. In the case of equal scores, this primary alignment is assigned arbitrarily.

Using SAMtools view v1.2 [8], the set of primary (uniquely highest scoring) alignments was obtained using parameters -F 256 (which removes non primary alignments) and -F 12 (which removes all reads that are not mapped and whose mate is not mapped; this primarily – but not exclusively – retains those reads mapping in a proper pair, i.e. those located on the same chromosome, one on either strand, orientating towards each other and spanning a reasonable insert size). The set of singleton reads (which map but have an unmapped mate) was obtained using SAMtools view with parameters -F 4 -f 8 -F 256. Finally, these two subsets were merged using Picard Tools [39] to create a file of uniquely mapped reads.

The tissue-specific transcriptome was assembled for this set of mapped reads using StringTie v1.2.3 [40] with default parameters, generating a corresponding GTF. In order to create a uniform, non-redundant set of transcripts for comparative purposes, these individual GTFs were then merged using StringTie --merge. StringTie was then re-run for each sample with parameters -G, -b and -e, now specifying not the reference annotation (ftp://ftp.ensembl.org/pub/release-81/gff3/ovis_aries/Ovis_aries.Oar_v3.1.81.gff3.gz, downloaded 18^th^ August 2015) but the merged GTF. Finally, gene-level expression estimates – comparable across samples produced by the same experimental protocol – were calculated using the R/Bioconductor package Ballgown [41].

By default, genes are assigned an ‘mstrg’ ID, a unique, StringTie-specific identifier. However, adjacent genes in the reference annotation may otherwise share an mstrg ID if reads map between them. This is not necessarily incorrect as multiple genes may be transcribed as a single operon, but it does introduce ambiguity into per-gene TPM estimates as there is not always a one-to-one correspondence of mstrg to Ensembl gene IDs. In these cases, we consider gene-level TPM to be identical for each gene assigned a single mstrg ID. That this occurs is because we retain the default StringTie parameter of -g 50 (minimum gap locus separation value = 50bp). In this case, StringTie will merge reads that map closer than 50bp in the same processing bundle, closing coverage gaps so as to increase the number of full-length structures possible for lowly expressed genes. This is a trade-off between sensitivity and specificity and is most pronounced in gene-dense regions: if coverage gaps were not filled, the assembly will be more fragmented (although there will be fewer erroneous merges).

The StringTie assembly is highly accurate with respect to the existing (Oar v3.1) annotation, successfully reconstructing almost all exon (96%), transcript (98%) and gene (99%) models (**S25 Table**). Nevertheless, StringTie also predicts many novel models (**S26 Table**), although in the absence of experimental verification, it is not easy to predict which are genuine, as opposed to stochastic noise in RNA processing or assembly artefacts [40]. The number of false positives is also likely exacerbated by the merger of both mRNA-Seq and total RNA-Seq data. The latter measures nascent (ongoing) transcription [42] and consequently has a larger proportion of retained introns arising from incompletely spliced pre-mature (nuclear) mRNA [43]. In any case, in the context of transcript annotation, false positives are easier to identify and correct than false negatives.

**Enrichment analyses**

For co-expression clusters containing > 50 genes and for the set of genes with higher expression in TxBF than Texel samples, GO term enrichment of each cluster was assessed using the R package topGO [44]. This utilises the ‘weight’ algorithm to account for the nested structure of the GO tree, with correction for multiple hypothesis testing intrinsic to the approach [45]. topGO requires a reference set of GO terms, built manually by obtaining the Oar v3.1 set from Ensembl BioMart v83 [46] and filtering to remove those with evidence codes NAS (non-traceable author statement) or ND (no biological data available). We further exclude from analysis those GO terms annotated to fewer than 50 genes in the genome, and any term where the observed number of genes annotated to it in the cluster does not exceed the expected number by 2-fold or greater. Given the GO tree is nested, the ‘depth’ of a GO term – its maximum distance from a parent term – was also determined using a custom script that queried the SUPERFAMILY database [47] (as the enrichment of ‘deeper’ terms – those with more specific functions – are of more immediate interest).

**Statistical analysis**

All statistical analyses were performed in R v3.2.2 [48]. Principal component biplots were made using the R packages ‘ggbiplot’ (https://github.com/vqv/ggbiplot) and ‘ggplot2’ [49].

**References**

1. Bush SJ, McCulloch MB, Summers KM, Hume DA, Clark EL. Integration of quantitated expression estimates from polyA-selected and rRNA-depleted RNA-seq libraries. BMC Bioinformatics*.* 2017;18(301).
doi:doi.org/10.1186/s12859-017-1714-9.

2. Trapnell C, Roberts A, Goff L, Pertea G, Kim D, Kelley DR, et al. Differential gene and transcript expression analysis of RNA-seq experiments with TopHat and Cufflinks. Nat Protocols. 2012;7(3):562-78. doi: 10.1038/nprot.2012.016.

3. Patro R, Mount SM, Kingsford C. Sailfish enables alignment-free isoform quantification from RNA-seq reads using lightweight algorithms. Nat Biotech. 2014;32(5):462-4. doi: 10.1038/nbt.2862.

4. Patro R, Duggal G, Kingsford C. Salmon: Accurate, Versatile and Ultrafast Quantification from RNA-seq Data using Lightweight-Alignment. bioRxiv. 2015.

5. Zhang Z, Wang W. RNA-Skim: a rapid method for RNA-Seq quantification at transcript level. Bioinformatics. 2014;30(12):i283-i92. doi: 10.1093/bioinformatics/btu288.

6. Bray NL, Pimentel H, Melsted P, Pachter L. Near-optimal probabilistic RNA-seq quantification. Nat Biotech. 2016;34(5):525-7. doi: 10.1038/nbt.3519.

7. Haas BJ, Papanicolaou A, Yassour M, Grabherr M, Blood PD, Bowden J, et al. De novo transcript sequence reconstruction from RNA-seq using the Trinity platform for reference generation and analysis. Nat Protocols. 2013;8(8):1494-512. doi: 10.1038/nprot.2013.084.

8. Li H, Handsaker B, Wysoker A, Fennell T, Ruan J, Homer N, et al. The Sequence Alignment/Map format and SAMtools. Bioinformatics. 2009;25(16):2078-9. Epub 2009/06/10. doi: 10.1093/bioinformatics/btp352.

9. Grabherr MG, Haas BJ, Yassour M, Levin JZ, Thompson DA, Amit I, et al. Full-length transcriptome assembly from RNA-Seq data without a reference genome. Nat Biotech. 2011;29(7):644-52. doi: 10.1038/nbt.1883.

10. Marçais G, Kingsford C. A fast, lock-free approach for efficient parallel counting of occurrences of k-mers. Bioinformatics. 2011;27(6):764-70. doi: 10.1093/bioinformatics/btr011.

11. Langmead B, Trapnell C, Pop M, Salzberg S. Ultrafast and memory-efficient alignment of short DNA sequences to the human genome. Genome Biology. 2009;10(3):R25. PubMed PMID: doi:10.1186/gb-2009-10-3-r25.

12. Mistry J, Finn RD, Eddy SR, Bateman A, Punta M. Challenges in homology search: HMMER3 and convergent evolution of coiled-coil regions. Nucleic Acids Research. 2013;41(12):e121. doi: 10.1093/nar/gkt263.

13. Finn RD, Coggill P, Eberhardt RY, Eddy SR, Mistry J, Mitchell AL, et al. The Pfam protein families database: towards a more sustainable future. Nucleic Acids Research. 2016;44(D1):D279-D85. doi: 10.1093/nar/gkv1344.

14. Camacho C, Coulouris G, Avagyan V, Ma N, Papadopoulos J, Bealer K, et al. BLAST+: architecture and applications. BMC Bioinformatics. 2009;10:421. Epub 2009/12/17. doi: 10.1186/1471-2105-10-421.

15. Boutet E, Lieberherr D, Tognolli M, Schneider M, Bairoch A. UniProtKB/Swiss-Prot. Methods Mol Biol. 2007;406:89-112. Epub 2008/02/22.

16. The UniProt Consortium. UniProt: a hub for protein information. Nucleic Acids Res. 2015;43(Database issue):D204-12. Epub 2014/10/29. doi: 10.1093/nar/gku989.

17. Altschul SF, Madden TL, Schäffer AA, Zhang J, Zhang Z, Miller W, et al. Gapped BLAST and PSI-BLAST: a new generation of protein database search programs. Nucleic acids research. 1997;25(17):3389-402. doi: 10.1093/nar/25.17.3389.

18. Rice P, Longden I, Bleasby A. EMBOSS: the European Molecular Biology Open Software Suite. Trends Genet. 2000;16(6):276-7. doi: 10.1016/S0168-9525(00)02024-2.

19. Wang L, Park HJ, Dasari S, Wang S, Kocher J-P, Li W. CPAT: Coding-Potential Assessment Tool using an alignment-free logistic regression model. Nucleic Acids Research. 2013. doi: 10.1093/nar/gkt006.

20. Fickett JW. Recognition of protein coding regions in DNA sequences. Nucleic Acids Research. 1982;10(17):5303-18. doi: 10.1093/nar/10.17.5303.

21. Fickett JW, Tung C-S. Assessment of protein coding measures. Nucleic Acids Research. 1992;20(24):6441-50. doi: 10.1093/nar/20.24.6441.

22. Wagner GP, Kin K, Lynch VJ. Measurement of mRNA abundance using RNA-seq data: RPKM measure is inconsistent among samples. Theory Biosci. 2012;131(4):281-5. Epub 2012/08/09. doi: 10.1007/s12064-012-0162-3.

23. Sultan M, Amstislavskiy V, Risch T, Schuette M, Dökel S, Ralser M, et al. Influence of RNA extraction methods and library selection schemes on RNA-seq data. BMC Genomics. 2014;15(1):1-13. doi: 10.1186/1471-2164-15-675.

24. Cui P, Lin Q, Ding F, Xin C, Gong W, Zhang L, et al. A comparison between ribo-minus RNA-sequencing and polyA-selected RNA-sequencing. Genomics. 2010;96(5):259-65. Epub 2010/08/07. doi: 10.1016/j.ygeno.2010.07.010.

25. Zhao W, He X, Hoadley KA, Parker JS, Hayes DN, Perou CM. Comparison of RNA-Seq by poly (A) capture, ribosomal RNA depletion, and DNA microarray for expression profiling. BMC Genomics. 2014;15:419. Epub 2014/06/04. doi: 10.1186/1471-2164-15-419.

26. Raz T, Kapranov P, Lipson D, Letovsky S, Milos PM, Thompson JF. Protocol Dependence of Sequencing-Based Gene Expression Measurements. PLoS ONE. 2011;6(5):e19287. doi: 10.1371/journal.pone.0019287.

27. Yanai I, Korbel JO, Boue S, McWeeney SK, Bork P, Lercher MJ. Similar gene expression profiles do not imply similar tissue functions. Trends in Genetics. 2006;22(3):132-8. doi: doi.org/10.1016/j.tig.2006.01.006.

28. Son CG, Bilke S, Davis S, Greer BT, Wei JS, Whiteford CC, et al. Database of mRNA gene expression profiles of multiple human organs. Genome research. 2005;15(3):443-50. doi: 10.1101/gr.3124505.

29. Uhlen M, Oksvold P, Fagerberg L, Lundberg E, Jonasson K, Forsberg M, et al. Towards a knowledge-based Human Protein Atlas. Nature biotechnology. 2010;28(12):1248-50. Epub 2010/12/09. doi: 10.1038/nbt1210-1248.

30. Pruitt KD, Tatusova T, Maglott DR. NCBI Reference Sequence (RefSeq): a curated non-redundant sequence database of genomes, transcripts and proteins. Nucleic acids research. 2005;33(Database Issue):D501-D4. doi: 10.1093/nar/gki025.

31. Khrunin AV, Filippova IN, Aliev AM, Tupitsina TyV, Slominsky PA, Limborska SA. GSTM1 copy number variation in the context of single nucleotide polymorphisms in the human GSTM cluster. Molecular Cytogenetics. 2016;9(1):30. doi: 10.1186/s13039-016-0241-0.

32. Yanai I, Benjamin H, Shmoish M, Chalifa-Caspi V, Shklar M, Ophir R, et al. Genome-wide midrange transcription profiles reveal expression level relationships in human tissue specification. Bioinformatics. 2005;21(5):650-9. Epub 2004/09/25. doi: 10.1093/bioinformatics/bti042.

33. Schug J, Schuller WP, Kappen C, Salbaum JM, Bucan M, Stoeckert CJ, Jr. Promoter features related to tissue specificity as measured by Shannon entropy. Genome Biol. 2005;6(4):R33. Epub 2005/04/19. doi: 10.1186/gb-2005-6-4-r33.

34. Julien P, Brawand D, Soumillon M, Necsulea A, Liechti A, Schütz F, et al. Mechanisms and Evolutionary Patterns of Mammalian and Avian Dosage Compensation. PLoS Biol. 2012;10(5):e1001328. doi: 10.1371/journal.pbio.1001328.

35. Huminiecki L, Lloyd A, Wolfe K. Congruence of tissue expression profiles from Gene Expression Atlas, SAGEmap and TissueInfo databases. BMC Genomics. 2003;4(1):31. doi:10.1186/1471-2164-4-31.

36. Andrews S. FastQC: a quality control tool for high throughput sequence data 2010. Available from: http://www.bioinformatics.babraham.ac.uk/projects/fastqc.

37. Bolger AM, Lohse M, Usadel B. Trimmomatic: a flexible trimmer for Illumina sequence data. Bioinformatics. 2014;30(15):2114-20. doi: 10.1093/bioinformatics/btu170.

38. Kim D, Langmead B, Salzberg SL. HISAT: a fast spliced aligner with low memory requirements. Nat Meth. 2015;12(4):357-60. doi: doi.org/10.1038/nmeth.3317.

39. The Broad Institute. Picard: a set of tools (in Java) for working with next generation sequencing data in the BAM format 2014. Available from: <http://broadinstitute.github.io/picard/>.

40. Pertea M, Pertea GM, Antonescu CM, Chang T-C, Mendell JT, Salzberg SL. StringTie enables improved reconstruction of a transcriptome from RNA-seq reads. Nat Biotech. 2015;33(3):290-5. doi: doi.org/10.1038/nbt.3122.

41. Frazee AC, Pertea G, Jaffe AE, Langmead B, Salzberg SL, Leek JT. Ballgown bridges the gap between transcriptome assembly and expression analysis. Nat Biotech. 2015;33(3):243-6. doi: 10.1038/nbt.3172.

42. Ameur A, Zaghlool A, Halvardson J, Wetterbom A, Gyllensten U, Cavelier L, et al. Total RNA sequencing reveals nascent transcription and widespread co-transcriptional splicing in the human brain. Nat Struct Mol Biol. 2011;18(12):1435-40. doi: 10.1038/nsmb.2143.

43. Zhang X, Rosen BD, Tang H, Krishnakumar V, Town CD. Polyribosomal RNA-Seq reveals the decreased complexity and diversity of the Arabidopsis translatome. PLoS One. 2015;10(2):e0117699. Epub 2015/02/24. doi: 10.1371/journal.pone.0117699.

44. Alexa A, Rahnenfuhrer J. topGO: Enrichment analysis for Gene Ontology 2010. Available from: <http://www.bioconductor.org/packages/release/bioc/html/topGO.html>.

45. Alexa A, Rahnenführer J, Lengauer T. Improved scoring of functional groups from gene expression data by decorrelating GO graph structure. Bioinformatics. 2006;22(13):1600-7. doi: 10.1093/bioinformatics/btl140.

46. Kinsella RJ, Kahari A, Haider S, Zamora J, Proctor G, Spudich G, et al. Ensembl BioMarts: a hub for data retrieval across taxonomic space. Database : the journal of biological databases and curation. 2011; Epub 2011/07/26. doi: 10.1093/database/bar030.

47. Gough J, Karplus K, Hughey R, Chothia C. Assignment of homology to genome sequences using a library of hidden Markov models that represent all proteins of known structure. J Mol Biol. 2001;313(4):903-19. Epub 2001/11/08. doi: 10.1006/jmbi.2001.5080.

48. R Core Team. R: A Language and Environment for Statistical Computing 2014. Available from: [http://www.r-project.org/](http://www.R-project.org).

49. Wickham H. ggplot2: Elegant Graphics for Data Analysis. New York: Springer-Verlag; 2009.
